# Genotoxicity Assessment in Occupational Health Personnel Exposed to Cytostatic Drugs in a Peruvian Hospital

**DOI:** 10.3390/genes17040418

**Published:** 2026-03-31

**Authors:** Luis Miguel Serquén López, Greta Milagros Mendoza Cornejo, Viviana Brigith Torres Merino, Blanca Pacheco Gonzales, Herry Lloclla Gonzales, Ricardo Leonidas de Jesús Vélez Chicoma

**Affiliations:** Research Center, Professional School of Environmental Engineering, César Vallejo University, Lambayeque 14000, Peru; greta.10.mili@gmail.com (G.M.M.C.); vivianatorresmrino@gmail.com (V.B.T.M.); ipachecogo01@ucvvirtual.edu.pe (B.P.G.); hlloclla@ucv.edu.pe (H.L.G.); rvelez@ucv.edu.pe (R.L.d.J.V.C.)

**Keywords:** genotoxicity, cytostatics, comet assay, tail percentage, tail moment

## Abstract

The use of cytostatic drugs for cancer treatment is currently the main weapon in the fight against cancer; however, prolonged exposure of healthcare personnel can cause adverse toxic effects. **Objective:** To determine the genotoxicity caused by exposure to cytostatic drugs, using the comet assay, in workers in the oncology department of a tertiary hospital in northern Peru. **Methodology:** Descriptive, quantitative, correlational, and cross-sectional study. The population consisted of two groups of workers: exposed (n = 40) and unexposed (n = 40). The alkaline lysis comet DNA technique was used on peripheral blood cells; tailing moment and tailing percentage indicators were evaluated. **Results:** Using nonparametric tests, the percentage and tail moment showed no significant differences, with *p* values of 0.8928 and 0.4675, respectively. The distribution observed in the group exposed to cytostatic drugs (pharmacists and pharmacy technicians) compared to the control group showed a normal distribution, with a tail moment of 8.29 vs. 3.03 and a percentage of tail of 37.12 vs. 23.24, respectively. Multivariate analysis showed that the tail moment variable was 11.56% greater in the group of pharmacists and pharmacy technicians (*p* = 0.0119) compared to the other participants. **Conclusions:** Although no significant difference was found, a trend toward a higher percentage and tail moment was observed in the group exposed to cytostatic drugs. Furthermore, the group of pharmacists and pharmacy technicians, compared to the other professions, showed significantly greater damage.

## 1. Introduction

Cancer is a disease that is becoming more prevalent, with 22 million new cases expected by 2022 and a projected increase to 35 million by 2050 [1]. Globally, around 19% of cancer cases are environmentally caused, and between 2% and 8% of all cancers are attributed to occupational factors [2]. However, the figures may be higher due to the low probability of defining the cause–effect relationship [3]. Globally, occupational exposure to xenobiotic agents is estimated to contribute significantly to mortality and disability-adjusted life years (DALYs), which in 2021 had a rate of 82.13 per 100,000 inhabitants [4]. On the other hand, more industrialized countries tend to have a higher number of occupational cancer cases. Some studies show that middle- and low-income countries have higher occupational exposure that causes cancer due to the lack of efficient regulations governing the marketing, use, and protective measures against carcinogens [5]. In Peru, it is estimated that 1.7% of cancers are environmentally caused [6].

In the healthcare sector, occupational cancer represents a critical challenge, where workers face unique risks from genotoxic agents such as ionizing radiation [7,8], antineoplastic drugs [9], and volatile organic compounds (VOCs), resulting in oxidative stress due to the production of reactive oxygen species (ROS), genotoxicity, and inflammation [10]. Chronic exposure to these agents induces direct genotoxic damage due to the presence of DNA adducts and single- and double-strand breaks, exceeding the DNA’s repair capacity. This manifests as chromosomal aberrations, micronuclei, and breaks in genetic material, which in the long term contribute to genomic instability and malignant transformation [11]. Thus, despite the widespread use of cytostatic drugs, which are essential in the treatment of neoplastic diseases, their non-selective mechanism of action affects not only tumor cells but also healthy cells, causing adverse effects [12,13]. Bladder cancer is the most closely related to this exposure [8], and it is also evident in 20–30% of those exposed to surface contamination detected at levels up to 634.2 ng/cm^2^ for ifosfamide [14].

It is important to determine the level of DNA damage, which is possible through tests such as the comet assay, micronucleus test, or *γ-H2AX* polymorphism [9,15]. The comet DNA test has proven to be versatile and the most widely used for this purpose [16,17].

Systematic scoping reviews analyzing 334 studies reinforce the use of the comet assay to assess DNA damage in occupational biomonitoring studies, highlighting the need for recent original studies to further reinforce this association and the use of safety measures to reduce risks [18].

In Europe, studies assessing DNA tailing damage in lymphocytes from exposed workers show average rates of 17–20%, with more than 50% of nuclei exhibiting “long tails,” which is higher than in control groups [19]. In Latin America, studies confirm occupational genotoxic damage with increases in comet and micronucleus indices. Nurses with higher exposure show higher tail moments (43.2 vs. 28.6) in buccal cells, even with low contamination [20]. However, in Latin America, data are scarce and fragmented, and in Peru, information on the genotoxic risk in high-complexity hospitals is practically nonexistent.

In a study of hospital workers in Italy, researchers examined 214 exposed individuals and 164 controls—oncology staff from seven hospitals—and found that the differences in the percentages of tail DNA were 15.88 ± 3.44 in the control group (CG) and 19.44 ± 4.51 in the exposed group (EG), and tail duration was 4.33 ± 1.28 in the CG and 6.06 ± 1.78 in the EG [21]. Analysis of the occupational effect in workers exposed to antineoplastic agents (305 exposed and 150 controls), measuring the same parameters with adjusted β values of 1.16, 0.17, 0.18, and 1.03 in the exposed group, found significant differences (*p* < 0.05) associating exposure with greater damage [22]. A study of professionals who administer antineoplastic agents—approximately 30 exposed and 30 controls—discovered DNA tailing at 10.35 ± 0.25 and 10.42 ± 0.18, with no significant differences or demonstrated genotoxic damage, focusing on oxidative stress and DNA damage [23]. In a population of oncology workers in Colombia, genotoxic damage was observed at 4.62 ± 1.477 in the exposed group and 2.41 ± 0.577 in the control group, with a significant difference (*p* < 0.001); occupational exposure was associated with increased comet and micronucleus damage indices; nurses with higher exposure showed higher tail moments (43.2 vs. 28.6) in buccal cells, even with low contamination [20].

The risk to these exposed personnel is exacerbated by factors such as non-compliance with safety regulations, poor implementation of personal protective equipment (PPE), and a lack of ongoing training [24]. At the same time, there is a growing demand for doctors, nurses, nursing technicians, pharmaceutical chemists, and pharmacy technicians involved in the administration of cytostatic drugs [25,26].

This study used the comet assay to evaluate cytogenetic damage in workers exposed and not exposed to cytostatic drugs. It identified the relationship between damage and exposure, and also compared the different professions exposed in a referral hospital in northern Peru.

## 2. Materials and Methods

### 2.1. Study Population

A descriptive, quantitative, correlational, and cross-sectional study was conducted at the Lambayeque Regional Hospital. The population consisted of 80 participants, divided into two groups: an exposed group (sample) and a control group (non-clinical). The exposed group included 40 oncology department workers who routinely handled cytostatic drugs (13 nurses, 11 nursing technicians, 6 pharmacy technicians, 6 pharmacists, 1 physician, and 3 oncologists). Conversely, the non-clinical group comprised 40 workers from the same hospital (1 lawyer, 2 administrators, 1 architect, 15 biologists, 5 bio-microbiologists, 2 accountants, 1 economist, 3 nurses, 4 engineers, 2 physicians, 1 clinical laboratory technician, and 3 medical technologists) without direct exposure to cytostatic drugs. The inclusion criteria were: aged between 18 and 65 years, in apparently good health, not using tobacco or being a smoker, and not having been exposed to ionizing radiation in the last six months. It is worth noting that all professionals who participated in the study did so voluntarily through informed consent.

### 2.2. Ethical Considerations and Sample Collection

The study was approved by the hospital’s ethics and research committee. All participants were informed in detail about the objectives and procedures of the study and signed a voluntary informed consent form. Data confidentiality was ensured by coding the samples and entering them into a database. A 5 mL sample of peripheral venous blood was obtained from each participant in EDTA tubes (Zhejiang Gongdong Medical Technology Co., Ltd., Huangyan, Taizhou, China)

### 2.3. Comet Assay

The comet assay was performed under alkaline conditions following the standard protocol [27]. Briefly, lymphocytes isolated from blood samples were embedded in low-melting-point agarose on microscope slides, lysed to remove membranes and proteins, and subjected to electrophoresis in an alkaline solution (pH > 13). Finally, the samples were neutralized and stained with a fluorescent DNA dye. The samples were blinded to the personnel performing the image analysis. One hundred cells per individual were analyzed using an epifluorescent microscope (CX-31; Olympus, Tokyo, Japan). DNA damage was quantified using Comet Score software (version 2.0.0.38.), recording the “percentage of DNA in the tail” and “tail moment” as parameters of genotoxicity [28].

### 2.4. Statistical Analysis

Data analysis was performed using the STATA software (version 14). Due to the behavior of the samples, nonparametric tests were used. The Wilcoxon test, *T*-test, and Mann–Whitney U test were used to compare the medians of DNA damage between the exposed and control groups. The Kruskal–Wallis test was used to compare damage levels among different occupational categories within the exposed group. A statistical significance level of *p* < 0.05 was considered.

## 3. Results

**Figure 1:**
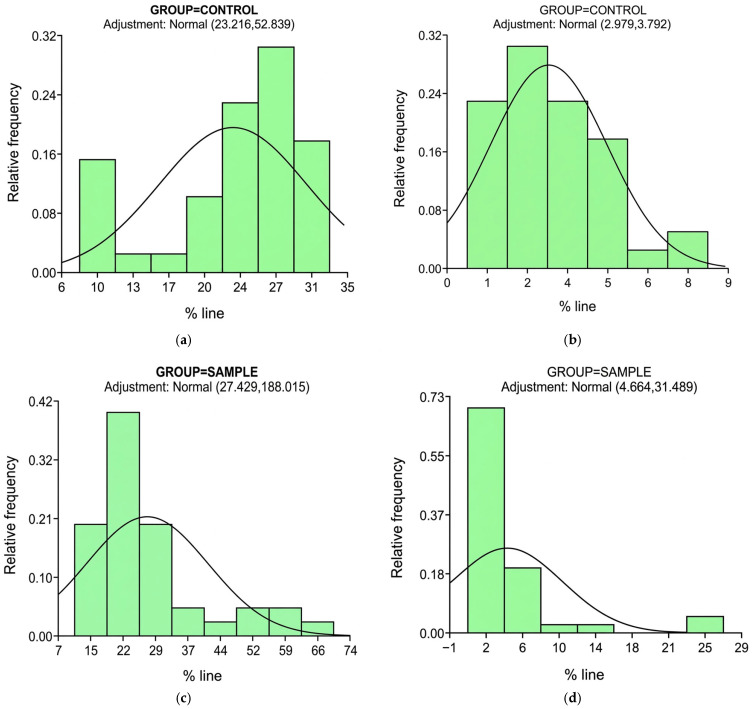
The graphs show the frequency of percentage of DNA in the tail and tail moment (**a**,**b**) belonging to the control group; (**c**,**d**) represent the sample group, where there is evidence of a tendency toward greater damage in that group. This shows that pharmaceutical chemists and pharmacy technicians are at greater risk of damage.

We observed a predominance of females in both groups, representing 82.5% (33/40) of the exposed group and 65% (26/40) of the control (non-clinical) group (Table 1). The average age was similar in both groups (37.58 ± 4.97 years for the exposed group and 37.08 ± 7.32 years for the control group). Furthermore, the most represented profession in the exposed group was nursing, while in the control group it was biology (Figure 1).

The Wilcoxon test was used to compare the medians of DNA damage between the exposed and control groups, and no differences were found considering the nonparametric behavior (*p* < 0.05). However, using the one-tailed independent-sample *T*-test and considering the parametric behavior of the data for both percentages and tail moment, *p* = 0.0456 and *p* = 0.395 were obtained, respectively (Table 2).

A multivariate analysis was performed, revealing 11.56% greater damage with a *p*-value of 0.0119 in the group of pharmaceutical chemists and pharmacy technicians compared to unexposed personnel (control) (Table 3); likewise, the distribution of the data shows a trend toward greater damage (Table 4).

## 4. Discussion

This study evaluated DNA damage in oncology professionals, 82.5% of whom were women and 32.5% of whom were nurses. Other authors highlight that women represent between 70% and 100% of the subjects analyzed [11,29]. This population of reproductive age becomes particularly vulnerable, with several studies reporting risks associated with spontaneous abortion (RR = 1.26–1.8) [30,31,32] and congenital malformations (RR = 1.76) [31]. Similarly, there is an increased risk associated with the direct handling of cytostatic drugs (OR = 2.5) [32] and an OR of 3.78 for spontaneous abortion in oncology nurses exposed to these agents [33]. These findings underscore the need to implement strict biosafety measures to minimize exposure and protect the reproductive health of healthcare personnel, especially considering the genotoxic nature of antineoplastic drugs and their ability to induce critical molecular alterations in human germ cells.

No significant differences are reported between the control group and the sample [23,34]. Similarly, other studies observed no correlation between prolonged exposure and detectable genetic alterations, using methodologies such as micronucleus analysis and the comet assay in different cohorts [35,36]. These results could be attributed to the proper use of personal protective equipment and improved working conditions. However, although no significant differences in genotoxic damage were observed, there may be greater susceptibility to oxidative stress in the exposed group [23]. Similarly, some authors suggest that within the exposed group there may be individual variability or differences in exposure [37].

Analyzing the group of exposed individuals, where a tendency toward greater damage is observed, pharmaceutical chemists and pharmacy technicians are included in this tendency to present cytotoxicity with a DNA percentage of 37.12 compared with 23.24 for the other professionals considered at risk (*p* = 0.0209), and a tail moment of 8.29 compared with 3.03 (*p* = 0.0355). Multivariate analysis confirmed these differences when comparing the occupational group with the control group, with 11.56% greater damage and a *p* = 0.0119. This is consistent with recent studies that highlight the risks for pharmaceutical chemists and pharmacy technicians related to the handling of cytostatics and oncological mixtures; the study could be expanded to other cancer centers in the region in order to statistically strengthen the results obtained [11,13,22,38].

A total of 95% of participants in the study regularly use PPE as a containment measure, while the remaining 5% are medical personnel who stated that they do not use PPE and although most of these healthcare workers see patients in their offices, they represent a high-risk group that requires specific awareness-raising initiatives. That is why awareness workshops are conducted with a focus on behavior-based safety, as it is geared towards providing more direct and participatory feedback from employers, where the focus is not only on sanctions or punishment, but also on fostering and promoting a culture of occupational safety and health. The preparation of oncological mixtures, as well as their administration, according to systematic reviews, confirms the relevance of biomonitoring to mitigate genotoxic risks [13,16]. Likewise, experimental studies reinforce the urgency of rigorous containment protocols, air quality control, and proper waste disposal [29,39].

This study did not include environmental monitoring; future studies should include the measurement of drug concentrations such as paclitaxel, cisplatin, Adriamycin, and cyclophosphamide, as well as 5-fluorouracil, which are the most commonly used drugs in the regional hospital’s oncology unit and require environmental monitoring. In turn, tests such as the Ames test would allow for the determination of exposure concentrations considered mutagenic in individuals with higher genotoxicity values. Studies such as the micronucleus test, chromosomal aberrations, and sister chromatid breaks would complement the findings.

The protective measures implemented to protect healthcare personnel handling cytostatic drugs include the use of closed system transfer devices (CSTDs), the strict use of personal protective equipment, and the protocols proposed by the institution for the management of chemical waste. Likewise, the results obtained from samples of individuals with a slight predisposition to exposure (pharmacy technicians and pharmaceutical chemists) were sent to the hospital’s occupational health department for continuous medical monitoring. This monitoring will allow for a review of their medical histories, family history, and lifestyle, in order to perform a series of tests using biomarkers and in vitro diagnostic (IVD) products, which will contribute to the timely identification of any cellular alterations resulting from cumulative exposure. Among other long-term measures, the architectural expansion of the Regional Hospital, as outlined in its master growth plan, must consider environments and equipment that ensure the protection of personnel, prioritizing those who interact with and are responsible for oncology mixtures.

Our laboratory, having the comet DNA test, will allow us to expand to studies in populations exposed to other types of xenobiotics, such as heavy metals, considering that, in the Lambayeque region, there is a rural population on the outskirts of the city that drinks from wells contaminated by groundwater.

## 5. Conclusions

The comet assay proved to be an effective tool for detecting genotoxic damage. One limitation of the study is the relatively small sample size, which could limit the statistical power to detect subtle differences in bivariate analyses. Future research should consider larger samples and multivariate analysis that includes cofactors and specific compliance with PPE practices during the preparation of oncology mixtures, as well as the monitoring of surfaces in clinical environments.

## Figures and Tables

**Table 1:** General data on workers in the oncology department and the control group.

Sex	Control Group n = 40	Sample Group n = 40
Female	26	33
Male	14	7
Age (mean ± DE)	37.08 ± 7.32	37.58 ± 4.97
**Duration of exposure to cytostatics (months)**	-	90 ± 48.66
**Use of PPE for handling cytostatic drugs (%)**	-	95%
**Profession**		
Lawyer *	1	-
Administrator *	2	-
Architect *	1	-
Biologist	15	-
Biologist–microbiologist	5	-
Public accountant *	2	-
Economist *	1	-
Nurse	3	13
Engineer *	4	-
Physician *	2	1
Oncologist	-	3
Pharmacist	-	6
Clinical laboratory technician	1	-
Nursing technician	-	11
Pharmacy technician	-	6
Medical technologist	3	-

* Non-clinical: personnel who do not have direct contact in the care and treatment of patients.

**Table 2:** Relationship of damage between exposed and non-exposed group.

	Wilcoxon Test			*T*-Test			
	Median		*p*-Value	Median		*p*-Value	Test
	Control (n = 40)	Sample (n = 40)		Control (n = 40)	Sample (n = 40)		
Percentage of DNA in the tail	25.37	22.21	0.8928	23.22	27.43	0.0912	Bilateral
Tail moment	2.81	2.99	0.4675	2.98	4.66	0.079	Bilateral
						0.0456	Unilateral
						0.0395	Unilateral

**Table 3:** Comparison between different professions exposed to cytostatic drugs.

	*T*-Test			Wilcoxon Test		
	Median		*p*-Value	Median		*p*-Value
	G1 * (n = 12)	G2 * (n = 68)		G1 * (n = 12)	G2 * (n = 68)	
**Percentage of DNA in the tail**	37.12	23.24	0.0209	34.42	24.17	0.093
**Tail moment**	8.29	3.03	0.0355	5.04	2.92	0.055

* G1: Group of pharmaceutical chemists and pharmacy technicians; G2: other professions in the study.

**Table 4:** Multiple linear regression analysis for exposure duration and exposed occupational groups.

Regression Coefficient	Estimation	Standard Error	Lower Limit (95%)	Upper Limit (95%)	T	*p*-Value	CpMallows	Variance Inflation Factor (VIF)
Constant	26.32	4.67	17.02	35.61	5.64	<0.0001		
Exposure time	−0.03	0.04	−0.12	0.05	−0.85	0.3997	3.72	2.56
Control	−2.78	4.1	−10.94	5.39	−0.68	0.5	3.46	3.27
QF and TF *	11.56	4.48	2.63	20.49	2.58	0.0119	9.64	2

* QF: pharmaceutical chemists; TF: pharmacy technicians/oncology solutions personnel.

## Data Availability

The datasets analyzed during the current study are available from the corresponding author upon reasonable request.
